# AuPt Alloy Nanostructures with Tunable Composition and Enzyme-like Activities for Colorimetric Detection of Bisulfide

**DOI:** 10.1038/srep40103

**Published:** 2017-01-04

**Authors:** Weiwei He, Xiangna Han, Huimin Jia, Junhui Cai, Yunlong Zhou, Zhi Zheng

**Affiliations:** 1 Key Laboratory of Micro-Nano Materials for Energy Storage and Conversion of Henan Province, College of Advanced Materials and Energy, Institute of Surface Micro and Nano Materials, Xuchang University, Henan 461000, P.R. China; 2 Wenzhou Institute of biomaterials and engineering, CNITECH, CAS, Zhejiang 325001, P.R. China; 3 Institute of biomaterials and engineering, Wenzhou Medical University, Zhejiang 325001, P.R. China

## Abstract

Tuning the enzyme-like activity and studying the interaction between biologically relevant species and nano-enzymes may facilitate the applications of nanostructures in mimicking natural enzymes. In this work, AuPt alloy nanoparticles (NPs) with varying compositions were prepared through a facile method by co-reduction of Au^3+^ and Pt^2+^ in aqueous solutions. The composition could be tuned easily by adjusting the molar ratios of added Pt^2+^ to Au^3+^. It was found that both peroxidase-like and oxidase-like activity of AuPt alloy NPs were highly dependent on the alloy compositions, which thus suggesting an effective way to tailor their catalytic properties. By investigating the inhibitory effects of HS^−^ on the enzyme-like activity of AuPt alloy NPs and natural enzyme, we have developed a method for colorimetric detection of HS^−^ and evaluation of the inhibiting effects of inhibitors on natural and artificial enzymes. In addition, the responsive ability of this method was influenced largely by the composition: AuPt alloy NPs show much lower limit of detection for HS^−^ than Pt NPs while Pt NPs show wider linear range than AuPt alloy NPs. This study suggests the facile way not only for synthesis of alloy nanostructures, but also for tuning their catalytic activities and for use in bioanalysis.

Nanostructured artificial enzymes, an emerging class of enzyme mimics, have received enormous interest due to their robust and efficient activities and potential applications ranging from biosensing and food processes to environmental protection and beyond^1^,^2^. Compared with natural enzymes, nanostructured artificial enzymes have several advantages, such as easy synthesis, facile storage and high catalytic stability against stringent conditions. Until now, a variety of nanomaterials, including metal oxides^3^,^4^,^5^, metal sulfides^6^,^7^, noble metals^8^,^9^,^10^,^11^, carbon^12^,^13^, and their combined nanostructures^14^,^15^ have been explored to exhibit enzyme-like activities. Because of the quantum size and surface effect, nanoparticles generally exhibit superior catalytic activity and intrinsic ability to generate or scavenge reactive oxygen species^16^,^17^,^18^. These properties are likely responsible for the mechanism by which the NPs mimic the catalytic activity of natural enzymes.

Metal NPs based enzyme mimetics have attracted particular attentions because of their defined mechanism, well-developed synthesis techniques, easy modification of surface and good bio-compatibility^19^,^20^,^21^. Several metal nanomaterials (e.g., Pt, Au, Pd, Ir) have been discovered for their intrinsic enzyme-like activities^8^,^9^,^10^,^11^. Among them, Pt family NPs recently have been found to exhibit multiple enzyme-like activities similarly to peroxidase, polyphenol oxidase, ferroxidase, catalase and SOD^9^,^22^,^23^,^24^. Bimetallic or multi-metallic nanostructures having unique properties dependent on structures and compositions can provide more versatile ways to optimize the enzyme-like activities than monometallic ones^25^,^26^,^27^,^28^. Zhang and coworkers have prepared Au@PtPd multi-component core/shell nanorods exhibiting tunable oxidase-like activity that was used for sensitive detection of Fe^2+^ ions^26^. Wang’s group has reported that PtCo bimetallic nanoparticles not only can be facilitated for magnetic separation, but also showed significantly improved oxidase-like activity for cancer-cell detection^27^. FeCo NPs were also found the much higher enzyme-mimic activity than other NPs-based peroxidase mimetics due to the synergistic effects^28^. The active and tunable enzyme-like activity may make bimetallic NPs potential valuable in bio-detections. In light of these findings and the unique properties of bimetallic NPs, a number of potential applications still remain to be explored that exploit the enzyme-mimicking functions of alloy NPs. For example, AuPt bimetallic nanostructures have exhibited intrinsic multiple enzyme-like activities, but most of these works involved AuPt bimetallic nanostructures rather than AuPt alloy nanoparticles^9^,^22^,^24^,^29^,^30^,^31^. The study on AuPt alloy nanoparticles for tailoring their chemical compositions to fine tune the oxidase and peroxidase-like activity and sensing capability for biodetection are limited. This knowledge will facilitate our understanding in depth the activity and applications of nanoenzymes.

In this study, we will take AuPt alloy NPs as an example to investigate their oxidase-like and peroxidase-like activities, and study the interaction with enzyme inhibitors for colorimetric detection. Especially, we will investigate systematically the effects of alloy compositions on both the enzyme-like activity and the sensing performance to enzyme inhibitors. We have developed a very simple yet effective way to prepare AuPt alloy nanostructures having porous structure and tunable composition. The oxidase-like and peroxidase-like activity of AuPt alloy NPs were found correlate closely with the compositions of Au and Pt. Furthermore, bisulfide (HS^−^) was selected as model to study its interactive behavior with enzymes or nanoenzymes because HS^−^ is an important gasotransmitter along with nitric oxide and carbon monoxide for biological signaling^32^. HS^−^ can inhibit effectively the peroxidase and oxidase like activity of AuPt bimetallic NPs. This inhibiting effect was same to the effect on nature enzyme horseradish peroxidase (HRP). The inhibitory degree was dependent on the concentration of HS^−^. Based on this finding, a facile method for colorimetric detection of HS^−^ was proposed with high sensitivity. The alloy of Au with Pt shows a large effect on the linear range and limit of detection for HS^−^.

## Results and Discussion

**Figure 1:**
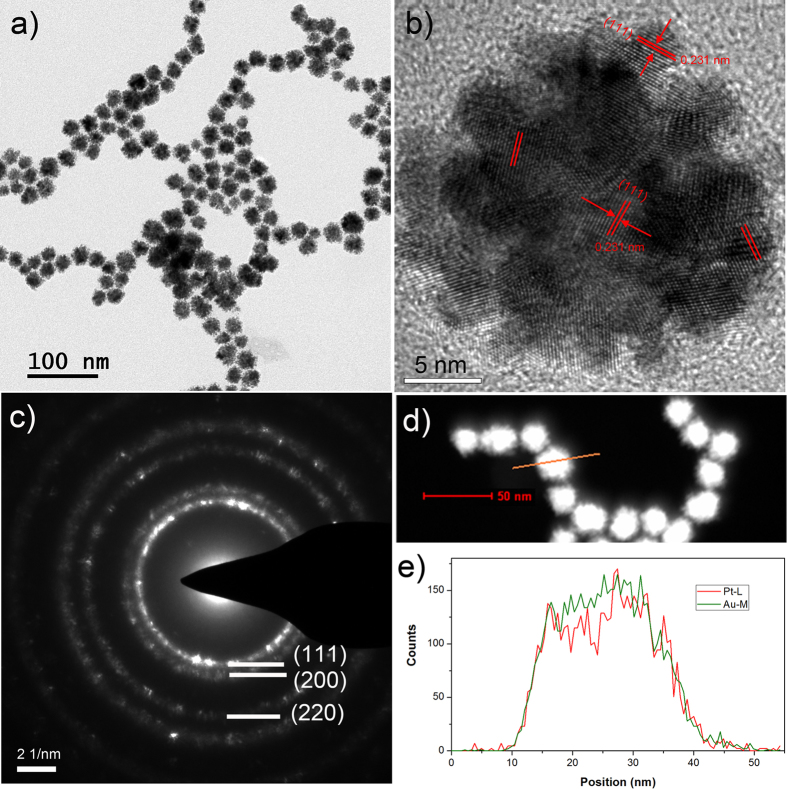
A typical (**a**) low-magnification TEM and (**b**) HRTEM image of AuPt nanostructures with an Au/Pt ratio of 1:1, (**c**) an ED pattern of a single particle in b, (**d**) a STEM HAADF image and (**e**) the STEM-EDX cross-section composition line profiles of the particle marked in (**d**).

AuPt alloy NPs were prepared by the co-reduction of AuCl_4_^−^ and PtCl_4_^2−^ with ascorbic acid in water. Figure 1 shows typical TEM images and the electron diffraction pattern of Au_0.5_Pt_0.5_ alloy nanostructures with an Au/Pt molar ratio of 1/1. The as-prepared AuPt alloy NPs have a spherical dendritic shape and a porous structure. They are well dispersed in solution. The average diameter of the Au_0.5_Pt_0.5_ alloy NPs was calculated from 50 random particles to be 23.6 ± 2.3 nm. The HRTEM further indicates the dendritic structure of the Au_0.5_Pt_0.5_ alloy NPs and the well-defined lattice planes (Fig. 1b). The calculated lattice spacing of the (111) facet is 0.231 nm, which falls between the values for pure Au (0.235 nm) and Pt (0.228 nm). This intermediate lattice spacing indicates the formation of an AuPt alloy structure. In the single particle, the orientation of the [111] plane differs in different part of the particle, indicating that the formation of the AuPt alloy may go through the attachment mechanism from smaller nanodots. The ED pattern shows that the diffraction spots are superimposed on the rings, which is consistent with the polycrystalline structure of single AuPt particle. The elemental distribution of Pt and Au in the nanoparticle was measured by STEM-EDX mapping of a single particle (Fig. 1d and e). The line profiles of Au and Pt in the selected particle show that Pt and Au are homogeneously mixed in the nanoparticle which further supports the formation of an AuPt alloy structure.

**Figure 2:**
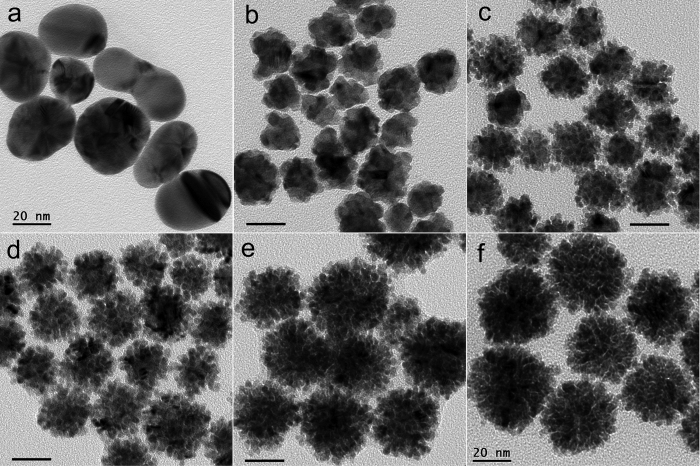
TEM images of (**a**) pure Au nanoparticles, AuPt bimetallic nanostructures with an Au/Pt molar ratio of (**b**) 3/1, (**c**) 1/1, (**d**) 1/3, (**e**) 1/6, and (**f**) Pt nanoparticles prepared under the same conditions. All the scale bars are 20 nm.

By changing the molar ratio of added Au^3+^/Pt^2+^, the morphology and the alloy compositions of AuPt nanoparticles can be controlled. Figure 2 shows that the shape of AuPt alloy NPs is highly dependent on the Au/Pt molar ratio. Pure Au NPs have irregular shapes with a solid structure and a smooth surface (Fig. 2a). The addition of Pt induces the formation of spherical NPs with a scraggly structure at a low Pt/Au ratio (0.33). Increasing the Pt/Au ratios from 0.33 to 6 produces a more condensed and porous structure, and obviously increases the particle size of AuPt alloy NPs. The particle sizes of particles with different Pt/Au ratios were calculated (see Supplementary Fig. S1). It was found that a higher Pt content produced larger particles, e.g. the diameter changed from 20 nm when the Pt/Au ratio was 1/3 to 40 nm when the Pt/Au ratio was 6/1. The presence of both Au and Pt in the particle also gave a larger particle size distribution (see Supplementary Fig. S1). AuPt alloy NPs with an Au/Pt ratio of 1/1 and 1/3 showed a narrower size distribution compared to other cases. These results indicated that the porous morphologies and particle size of AuPt alloy NPs can be subtly controlled by adjusting the Au/Pt ratio. Why such a simple method can produce uniform AuPt alloy NPs with dendritic structures? The reasons may be due to that the high bond dissociation energies and the large lattice mismatch (3.8%) between Au and Pt tend to favor the island growth mode^33^. In addition, the residual ascorbic acid and oxidized product, e.g. dehydroascorbic acid, may play a protective role in regulating the growth since no additional surfactant was added during the AuPt formation process^34^.

**Figure 3:**
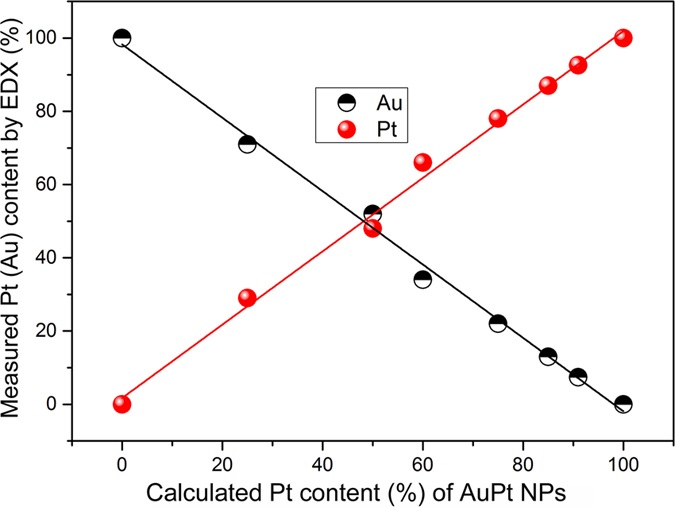
The measured Pt or Au content in AuPt nanostructures by EDX analysis as a function of the calculated Pt content (%) in AuPt nanostructures.

Apart from the morphology, varying the amount of added Pt^2+^/Au^3+^ also greatly influenced the alloy content in AuPt alloy NPs. The elemental Au and Pt content in prepared AuPt alloy NPs were measured by energy dispersive X-ray analysis (Fig. 3). The measured Pt content linearly increased and the Au content linear decreased with the increasing addition of Pt^2+^ from 0 to 100%. The two linear relationships have a slope near 1.0 or −1.0 which indicates a complete reduction of Au^3+^ and Pt^2+^ to form the AuPt alloy NPs. The linear relationship also reflects that the alloy composition can be tuned by changing the amount of Pt^2+^/Au^3+^ that is added.

**Figure 4:**
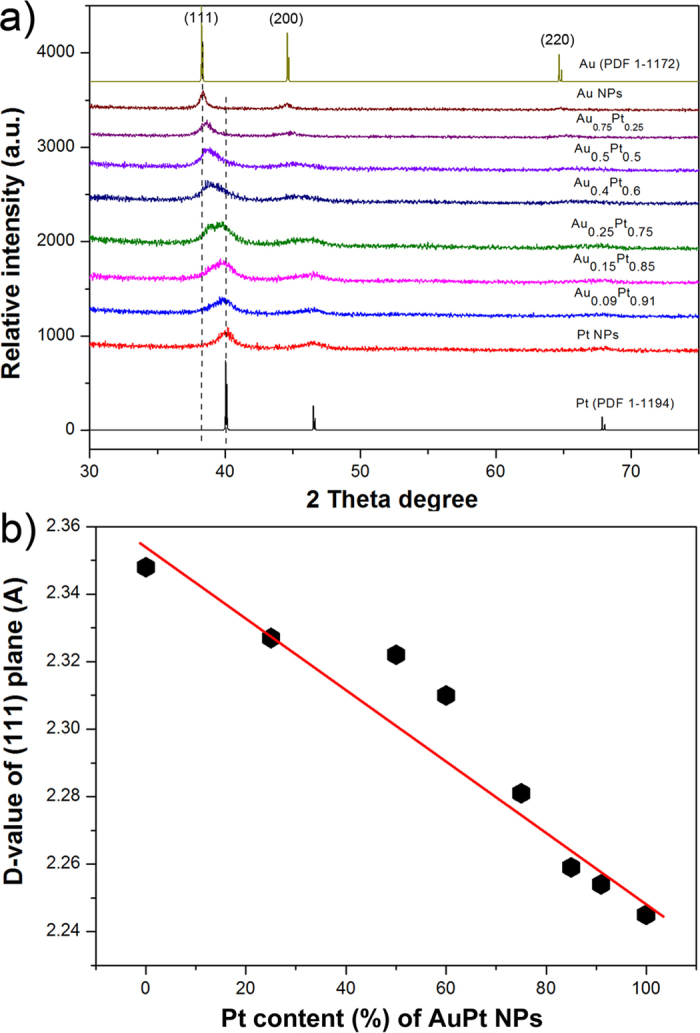
(**a**) XRD patterns of prepared Au NPs, Pt NPs and AuPt alloy nanostructures with varying compositions, (**b**) the plot of d-value variation of the (111) plane versus the Pt content in the AuPt nanostructures.

The formation of AuPt alloy NPs was also verified from UV-Vis absorption spectroscopy (see Supplementary Fig. S2). The pure Au NPs show a typical absorption band at 520 nm due to their surface plasmonic resonance property. The formation of bimetallic NPs as Pt is added leads to a less evident absorption band which disappeared gradually on higher Pt loading. The disappearance of this band indicates the formation of an alloy structure. The XRD patterns further confirmed the formation of AuPt alloy (Fig. 4). The diffraction peaks from the Au, the Pt and the AuPt NPs with various Au/Pt ratios indicates that all of the samples have a face center cubic phase. The 2θ degree of diffraction peaks (111) and (200) from the AuPt NPs fall between the corresponding 2θ degree in the Au and Pt NPs. These phases are gradually shifted upon increasing the Au/Pt ratio which indicates the change of alloy degree. The d-value of plane (111) was calculated from the XRD pattern by the Debye-Scherrer equation:


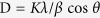


where K is the Scherrer constant, *λ* is the X-ray wavelength, *β* is the line broadening at half the maximum intensity (FWHM), and *θ* is the Bragg angle. The d-values of the AuPt NPs at various Au/Pt ratios all fell between the Au (111) of 0.235 nm and the Pt (111) of 0.224 nm. They show a quasi-linear relationship with the calculated Pt/(Au + Pt) ratio. Therefore, the degree of alloying and the crystal structures of the AuPt NPs are closely correlated to the Au/Pt ratio of added salts.

**Figure 5:**
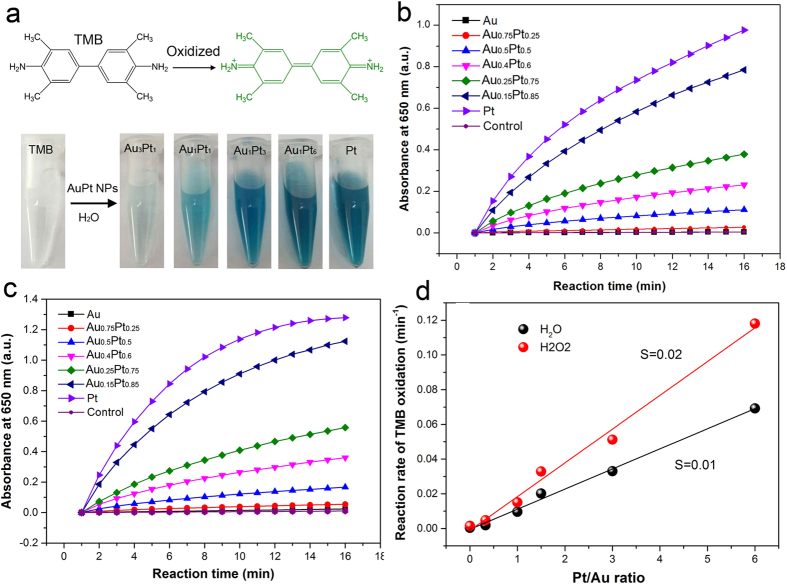
(**a**) Color evolution of TMB oxidation in the absence of hydrogen peroxide catalyzed by different NPs. The absorbance change at 650 nm as a function of reaction time during the TMB oxidation catalyzed by different catalysts in the absence (**b**) and in the presence (**c**) of hydrogen peroxide, (**d**) the reaction rate of TMB oxidation in water and in hydrogen peroxide is dependent on the Au/Pt ratio of the AuPt nanoalloy.

Au and Pt nanoparticles have achieved activities similar to those of enzymes in some reactions. For example, small Pt or Au NPs (e.g. <5 nm) have been shown to mimic multiple enzymes (i.e. peroxidase, oxidase, catalase and SOD) for a variety of applications^8^,^9^. It would be interesting, then, if varying the alloy composition of the AuPt alloy nanostructures could tune the enzyme-like activities of these catalysts. For proof of this concept, the AuPt alloy NPs with varied composition were subjected to reaction conditions typical for peroxidase and oxidase enzymes and the activities of the NPs were compared. 3,3,5,5-Tetramethylbenzidine (TMB) is a chromophoric substrate commonly used in nano-peroxidase mimetic studies and was chosen as a substrate to be oxidized in this studies. Pt NPs and AuPt NPs with various Au/Pt ratios can quickly catalyze the oxidation of TMB to produce a typical blue color either in the presence or absence of hydrogen peroxide (Fig. 5a, color changes in H_2_O_2_ not shown). Control reactions in the absence of AuPt alloy NPs were performed and showed negligible color changes over the same time period, implying that the AuPt alloy NPs, similar to natural oxidase and peroxidase, are responsible for the oxidation of TMB. While previous reports have shown that Au NPs have peroxidase-like activity^8^, the Au NPs herein do not mimic peroxidases or oxidases. This may be due to the larger particle size of Au NPs formed in this work (>30 nm). Figure 5a–c show the activities of the AuPt alloy NPs used in this reaction. It was found that the catalytic activities of the AuPt alloy NPs increased with an increasing Pt/Au ratio. The reaction rates of TMB oxidation both in the absence and in the presence of hydrogen peroxide were calculated and are shown in Fig. 5d. The linear relationship between the reaction rates and the AuPt alloy composition confirms that the rates increase with increasing Pt/Au ratio. It is important to note that the oxidation rate of TMB in 0.67 mM H_2_O_2_ is twice as fast as when the reaction is run in water. These results indicate that the alloy composition can affect the peroxidase-like and oxidase-like activities of AuPt NPs in the oxidation of TMB.

The apparent kinetic parameters of the AuPt nanostructures as oxidase mimetics for TMB oxidation in the absence of H_2_O_2_ were determined. Typical double-reciprocal plots of 1/ν vs. 1/[S] were constructed and fitted to the Michaelis-Menten equation to calculate the Michaelis constant (*K*_*m*_) and the maximal reaction velocity (*V*_*max*_) for the oxidation of TMB (see Supplementary Fig. S3). The values of both *K*_*m*_ and *V*_*max*_ were found to be highly dependent on the Pt content in the AuPt alloys. A higher Pt content led to an increase of *K*_*m*_ and *V*_*max*_. In other words, the AuPt alloy NPs having a higher Au content gave lower *K*_*m*_ and *V*_*max*_ values. In natural enzymes, *K*_*m*_ is an indicator of the affinity between the enzyme and the substrate where a lower *K*_*m*_ value indicates a higher affinity and catalytic activity. In the case of the nanostructure enzyme mimetics, the *K*_*m*_ value is often used to compare the enzyme-like performance of the nanostructures^4^,^5^,^6^. It was found that the AuPt alloy NPs oxidase mimetics with a lower *K*_*m*_value were accompanied by lower reaction rates in the oxidation of TMB, indicating the higher affinity of AuPt alloy NPs with substrate than that of Pt NPs. Generally, the specific affinity between substrate and inorganic nanoparticles is much weaker than between substrates and natural enzymes^35^. This work indicates that the use of *K*_m_ should be critically cautious to consider for evaluating the enzyme-like capabilities of inorganic nanostructures.

**Figure 6:**
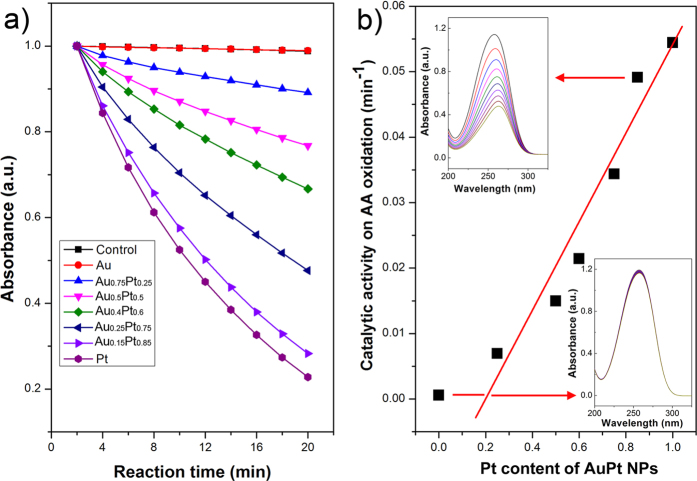
The effect of alloy composition on the catalytic activity of AuPt NPs toward ascorbic acid oxidation. (**a**) The normalized absorbance at 257 nm as a function of reaction time after the addition of Au NPs, Pt NPs and AuPt alloy NPs with various compositions. (**b**) The dependence of AA oxidation rates on Pt content in the AuPt NPs. Insets in (**b**) show the evolutions of the absorption spectra of AA over time for Au NPs and for Au_0.15_Pt_0.85_ alloy NPs.

Ascorbic acid (AA) is an essential nutrient and a well-known antioxidant that protects other important biological structures against oxidative damage by many oxidants. Although AA is slowly oxidized by oxygen, the oxidation can be accelerated by the addition of ascorbic acid oxidase (AAO). The intermediate ascorbyl radical is an indicator in this oxidation. In our previous work, we found that Au@Pt core-shell nanostructures behave similarly to AAO and can accelerate the oxidation of AA and reduce their antioxidant ability^24^. The AuPt alloy NPs also showed the intrinsic ability to quickly oxidize AA (Fig. 6). The oxidation of AA to ascorbyl radical is accompanied by a decrease in the characteristic absorption of AA near 260 nm. UV-Vis spectroscopy was used to measure this absorption band in order to study the reaction kinetics of the oxidation of AA (Fig. 6 insets). AA alone or AA in the presence of Au NPs reacted slowly by dissolved oxygen during the reaction period. However, the oxidation of AA was significantly accelerated upon the addition of Pt NPs and AuPt NPs with various compositions. A clear composition-dependence on catalytic oxidation of AA was observed. A higher Pt/Au ratio in the alloy NPs resulted in a faster AA oxidation. The oxidation rates of AA using different catalysts were calculated (Fig. 6b). The catalytic activities on AA oxidation were linearly proportional to the Pt content in the AuPt alloy NPs. Our experimental results are in remarkable agreement with previous calculations by Wu and coworkers^36^. The mechanisms of the oxidase-like activity of noble metals and their alloys have been studied by using density functional theory calculations. The alloy of Au with Pt decreases the oxidase-like reactivity of Pt due to higher activation energy barriers and positive reaction energies resulted from the addition of high Au content. Our experimental results suggest an effective way to tailor the AAO-like activity of Pt NPs and to diminish the antioxidant potential of AA by changing the elemental composition of the AuPt alloy NPs.

From Figs 5 and 6, it was found the same composition dependence of catalytic activities of AuPt alloy NPs in different catalytic reactions: a higher gold percentage in AuPt alloy NPs leads to more reduction of catalytic activities. This tendency was well consistent with previous reports on AgPt and PdPt alloy nanoparticles^26^,^37^. It was recognized that electronic structure of metal NPs plays crucial role in influencing their catalytic activity. Take Pt based bimetallic alloy as example, it has been established that alloying Pt with the 3d-transition metals can tune the d-band center position and consequently change their catalytic performance^38^. Herein, we proposed that the alloying Pt with Au may change the electronic structure of Pt and the electronic property was influenced largely by the Au content in alloy, which in turn affected the catalytic performance. This hypothesis may require further information for the the electronic structure change of AuPt alloy with varying composition by theoretical calculation.

**Figure 7:**
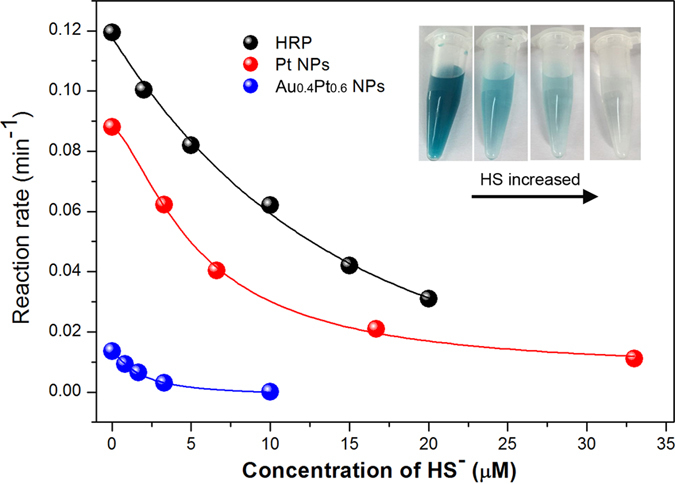
Concentration dependent effect of HS^−^ on inhibiting the activities of enzyme HRP, and Pt or Au_0.4_Pt_0.6_ alloy nanoparticles. Insets show photographs of TMB oxidation catalyzed by AuPt NPs as the concentration of HS^−^ increases.

Hydrogen sulfide has been identified as an important biological molecule with diverse functions in gaseous signaling and pathophysiological^39^. In addition, H_2_S can also interact with biochemical molecules or species, such as cytochrome C oxidase, NO and reactive oxygen species^40^,^41^, consequently affect their bioactivities. Because H_2_S is a weak acid, about 80% of H_2_S exists as monoanionic HS^−^ under physiological conditions. HS^–^ is highly reactive anion, as it is more reality oxidized than H_2_S. It is of biological importance to be able to detect HS^−^ and to understand the interactions this molecule has with other biological structures. In this work, we found that HS^−^ ions could significantly inhibit the peroxidase-like and oxidase-like activities of AuPt NPs, similarly to the effect on the activity of enzyme horseradish peroxidase (HRP). This, in turn, provided an efficient way to colorimetrically detect HS^−^ ions. Compared to the control experiment without HS^−^ ions (Fig. 7), the addition of HS^−^ inhibits the color evolution of TMB oxidation catalyzed by HRP, Pt NPs or AuPt NPs either in the presence or absence of hydrogen peroxide. The UV-Vis spectral evolution of TMB oxidation over time in the absence of SH^−^ and in the presence of SH^−^ is shown in Supplementary Fig. S4. The oxidation rates of TMB catalyzed by NPs and HRP in the absence and presence of HS^−^ were calculated. The inhibitory degree dependent on the concentration of HS^−^ was found for either natural enzyme HRP or nanoenzyme mimetic Pt and AuPt NPs (Fig. 7). They showed the same trend: a higher concentration of disulfide leads to a greater reduction in TMB oxidation rate. In addition, they may have the similar inhibitory mechanisms. HS^−^ can chemically bind with the metal ions of active site in HRP while HS^−^ may poison the active surface of the Pt or the AuPt NPs by metal-sulfur bond, which consequently results in a reduction of catalytic activity. It is important to note that the alloy with Au is required for good detection of HS^−^. For example, the AuPt NPs show a 4 times lower limit of detection for HS^−^ than pure Pt NPs. A concentration of 0.83 μM SH^−^ could almost completely inhibit the AuPt NPs used to catalyze the oxidation of TMB while a concentration of 3.3 μM SH^−^ was needed to inhibit the oxidation of TMB with Pt NPs. In contrast, the Pt NPs (3.3 μM to 33 μM) showed a wider linear range for detection of hydrosulfide than that of AuPt NPs at Au/Pt of 2/3 (0.83 μM to 10 μM). By using the enzyme-like activity of AuPt NPs, we demonstrated that it is feasible to not only fabricate a colorimetrical method for detection of HS^−^, but also change the sensing performance to HS^−^ by altering the alloy compositions.

**Figure 8:**
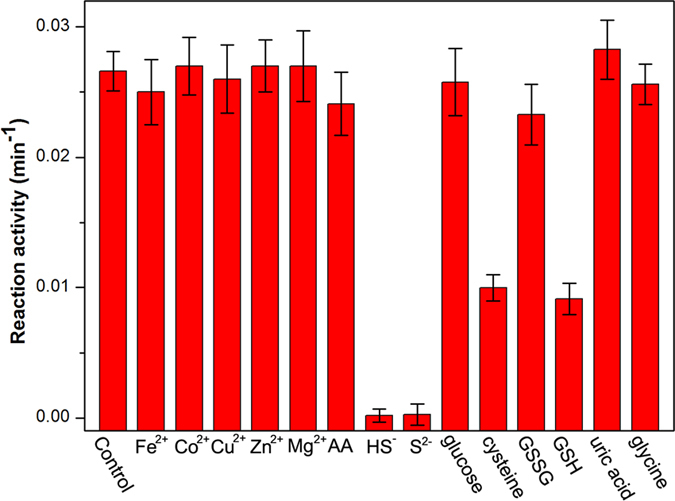
Selectivity for detection of bisulfide by employing the oxidase-like activity of Au_0.4_Pt_0.6_ alloy nanoparticles. The error bars represent the standard deviation of 3 measurements.

We further tested the selectivity of proposed method for detection of bisulfide. Several metal ions (Fe^2+^, Cu^2+^, Co^2+^, Zn^2+^ and Mg^2+^), sulfide ions (S^2−^), cysteine, GSH, glycine, AA, GSSG, glucose and uric acid, they are often-considered species in biological system, were selected to investigate their interference on the detection of bisulfide. The concentrations of all the interference substances were 20 μM, 4 times as high as HS^−^ and S^2−^ (5 μM). The result was displayed in Fig. 8. Metal ions (Fe^2+^, Cu^2+^, Co^2+^, Zn^2+^ and Mg^2+^), AA, glucose, GSSG, uric acid and glycine showed negligible effects on inhibiting the oxidase-like activity of Au_0.4_Pt_0.6_ alloy NPs. In contrast, sulfide ions, ionized product of HS^−^, can inhibit significantly the catalytic activity as same as bisulfide, while cysteine and GSH cause considerable reduction of catalytic activity. These results indicated that the method was sensitive to HS^−^ and S^2−^, but the chemical species with thiol-group may cause some interference.

For testing the feasibility of the method in practical samples, the determination of bisulfide in spiked human blood serum was performed. The serum samples were spiked with desirable amounts of bisulfide, and their inhibitory effect on the oxidase-like activities of Pt NPs and Au_0.4_Pt_0.6_ alloy NPs were evaluated (see Supplementary Fig. S5). Compared with the control in water, the un-spiked serum reduced slightly the catalytic activity for both Pt NPs and AuPt alloy NPs. The spiked serum samples with different concentrations of bisulfide (1–3 μM) exhibited distinct behavior for inhibiting the oxidase-like activity of Pt and AuPt alloy NPs. A linear and sensitive response was observed for AuPt alloy NPs while HS^−^ less than 3 μM cause unobvious inhibition for Pt NPs, indicating the higher detection capability of AuPt alloy NPs than Pt NPs. As we calculated above, the alloying Pt with Au gradually changed the electronic structure and decreased the K_m_, the lower K_m_ of AuPt alloy NPs than pure Pt NPs indicated the higher affinity which may result in lower limit of detection. These results indicated this method based on the oxidase-like activity of AuPt alloy NPs can be applied to bisulfide (sulfide) detection in real sample. Furthermore, it was expected that the inhibitors of natural antioxidants for nanomaterial mimics can mirror natural enzymes. This may provide a facile route to investigate the interaction between inhibitors and natural enzymes. Many other inhibitors are being investigated for their inhibitory activity and mechanism by using NPs enzyme mimics.

## Conclusions

AuPt alloy nanostructures with tunable compositions were synthesized through a facile method in aqueous solution by adjusting the molar ratio of added Pt^2+^ to Au^3+^. This method may be a cost-effective and environmentally-friendly way to prepare other binary or multicomponent metal nanostructures. AuPt alloy nanoparticles were verified to exhibit intrinsic peroxidase-like and oxidase-like activities that were highly dependent on the alloy compositions of AuPt NPs. This provides an effective way to tailor the enzyme-like properties of nanoparticles. By studying the inhibiting effect of bisulfide on the catalytic activities of enzyme HRP and AuPt NPs, we developed a platform that can be used for colorimetrical detection of bisulfide and sulfide to evaluate the inhibiting effect of inhibitors on natural enzymes and nanoenzymes. This method exhibits high sensitivity and selectivity to bisulfide (and its ionized product, sulfide) against selected metal ions and non-thiol containing biologically relevant molecules, yet biothiols may result in considerable interference. In addition, the sensing performance for HS^−^ can be tailored by changing the compositions of AuPt. Since many biological and chemical inhibitors can reduce the activity of natural enzymes, our results will also facilitate the investigation of biochemical interactions between enzymes and their inhibitors.

## Methods

### Chemicals

Chlorauric acid (HAuCl_4_∙3H_2_O), potassium tetrachloroplatinate (II) (K_2_PtCl_4_), cetyltrimethylammonium bromide (CTAB), hydrogen peroxide, L-ascorbic acid (AA), 3,3′,5,5′-tetramethylbenzidine (TMB), cysteine, L-glutathione reduced (GSH), L-glutathione oxidized (GSSG), horseradish peroxidase and sodium hydrosulfide (NaHS) were purchased from Alfa Aesar. FeSO_4_∙6H_2_O, Co(NO_3_)_2_∙6H_2_O, ZnSO_4_, CuSO_4_, Mg_2_SO_4_, Na_2_S∙9H_2_O, glucose, uric acid and glycine were obtained from Sinopharm Chemical Reagent Co., Ltd. Milli-Q water (18 MΩ cm) was used in the preparation of all solutions.

### Synthesis of Au, Pt and AuPt nanostructures

AuPt nanostructures were prepared by mixing desirable volumes of 17 mM AuCl_4_^−^ and 24 mM PtCl_4_^2−^ in 2 mL H_2_O (according to the calculated Au/Pt molar ratio in the bimetallic alloy from 3/1, 1/1, 1/1.5, 1/3, 1/6 to 1/10). A 40 μl 0.1 M AA solution (ratio of AA/(Au^3+^ + Pt^2+^) = 10) was added into the homogenous solution and was shaken vigorously. The solution was placed in a 30 °C water bath for 2 hours. The color became dark brown which suggested the formation of AuPt bimetallic NPs. Then, 0.1 mL 0.1 M CTAB was added into above solutions. The same procedure was used to prepare pure Au and Pt nanoparticles except without the addition of either PtCl_4_^2−^ or AuCl_4_^−^. The samples were purified by centrifugation at 12000 rounds per min for 10 min and the precipitation was re-dispersed in water. We named here the Au_x_Pt_1-x_ alloy NPs as Au_0.75_Pt_0.25_, Au_0.5_Pt_0.5_, Au_0.4_Pt_0.6_, Au_0.25_Pt_0.75_, Au_0.15_Pt_0.85_ and Au_0.09_Pt_0.91_ alloy NPs, respectively, according to the added Au/Pt ratio.

### Characterization

UV-visible absorption spectra were obtained using a UV-VIS-NIR Spectrometer (Varian Cary 5000) and a matched quartz cuvette with a path length of 1 cm. The crystal structures of the AuPt alloy nanoparticles were characterized by X-ray diffraction (XRD, Bruker D8 Advance diffractometer) using monochromatized Cu Kα radiation (λ = 1.5418 Å). Transmission electron microscopy (TEM) images were captured on a Tecnai G^2^ F20 U-TWIN electron microscope with an accelerating voltage of 200 kV. High-resolution TEM (HRTEM), selected-area electron diffraction and energy dispersive X-ray spectrometry were performed using the same microscope. Elemental composition and element distribution was identified by the spectrum-imaging method using a dedicated STEM and energy dispersive X-ray spectroscopy under a high-angle annular dark field (HAADF) mode. TEM analysis samples were prepared by adding a droplet of colloidal solution onto a standard holey carbon-coated copper grid and allowing it to air dry.

### Peroxidase-like and oxidase-like activities measurements

The reaction kinetics for the catalytic oxidation of TMB or AA were studied by recording absorption spectra at 2 min intervals in the scanning kinetics mode. Unless otherwise noted, reactions were performed at room temperature, with a fixed amount of AuPt nanoparticles, and with different concentrations of TMB or AA. For the catalytic oxidation of TMB in the presence of hydrogen peroxide, 20 μL of 20 mM TMB solution and 20 μL of 0.1 M H_2_O_2_ were added to 3 mL of H_2_O. To this solution, a suspension of 25 μL AuPt NPs (with different Au/Pt ratio) or Au or Pt NPs was added to initiate the oxidation of TMB. The reaction was also performed in the absence of hydrogen peroxide. Similarly, the catalytic oxidation of AA was initiated by adding 25 μl AuPt NPs into a 3 mL 0.1 mM aqueous solution of AA. The apparent kinetic parameters for the oxidation of TMB were calculated using the Michaelis-Menten equation υ = V_max_ × [S]/(K_m_ + [S]), where υ is the initial velocity, V_max_ is the maximal reaction velocity, [S] is the concentration of substrate and K_m_ is the Michaelis constant.

The inhibitory effect of bisulfide on the enzyme-like activity of AuPt NPs and enzyme HRP was evaluated by addition of 1.3 μg ml^−1^ AuPt NPs or 1.0 μg/mL HRP to a mixture containing 20 μL 20 mM TMB, 20 μl 0.1 M H_2_O_2_ and bisulfide at different concentrations in 3 ml H_2_O, and then the reaction process was monitored at 2 min intervals. The inhibiting effects of interference substances (Fe^2+^, Cu^2+^, Co^2+^, Zn^2+^, Mg^2+^, AA, glucose, GSSG, uric acid, glycine, cysteine and GSH) on the oxidase-like activity of AuPt alloy NPs were studied using the same experimental procedure except for their concentrations were fixed at 20 μM. To test the feasibility of the method in practical application, the human blood serum was obtained from hospital (Xinhua Hospital of Xuchang) and used as received. The blood serum samples were spiked with 1, 2 and 3 μM bisulfide, respectively. Then the spiked and unspiked serum samples were measured for their inhibitory effect on the enzyme-like activity of NPs by the procedure described above.

## Additional Information

**How to cite this article**: He, W. *et al*. AuPt Alloy Nanostructures with Tunable Composition and Enzyme-like Activities for Colorimetric Detection of Bisulfide. *Sci. Rep.* **7**, 40103; doi: 10.1038/srep40103 (2017).

**Publisher's note:** Springer Nature remains neutral with regard to jurisdictional claims in published maps and institutional affiliations.

## Supplementary Material

Supplementary Information
