# Supplementary material for: AuPt Alloy Nanostructures with Tunable Composition and Enzyme-like Activities for Colorimetric Detection of Bisulfide
Source: Sci Rep. 2017 Jan 4;7:40103. doi: 10.1038/srep40103 (PMC5209660; doi:10.1038/srep40103)
Supplement: Supplementary Information [file srep40103-s1.doc]

*Supplementary information:*

AuPt Alloy Nanostructures with Tunable Composition and Enzyme-like Activities for Colorimetric Detection of Bisulfide

Weiwei He^1^,*, Xiangna Han^1^, Huimin Jia^1^, Junhui Cai^1^, Yunlong Zhou^2,3^,*, Zhi Zheng^1^

^1^Key Laboratory of Micro-Nano Materials for Energy Storage and Conversion of Henan Province, College of Advanced Materials and Energy, Institute of Surface Micro and Nano Materials, Xuchang University, Henan 461000, P. R. China

^2^Wenzhou Institute of biomaterials and engineering, CNITECH, CAS, Zhejiang 325001, P. R. China

^3^Institute of biomaterials and engineering, Wenzhou Medical University, Zhejiang 325001, P. R. China

*Corresponding Author: heweiweixcu@gmail.com (W. H.); zhouyl@wibe.ac.cn (Y. Z.)


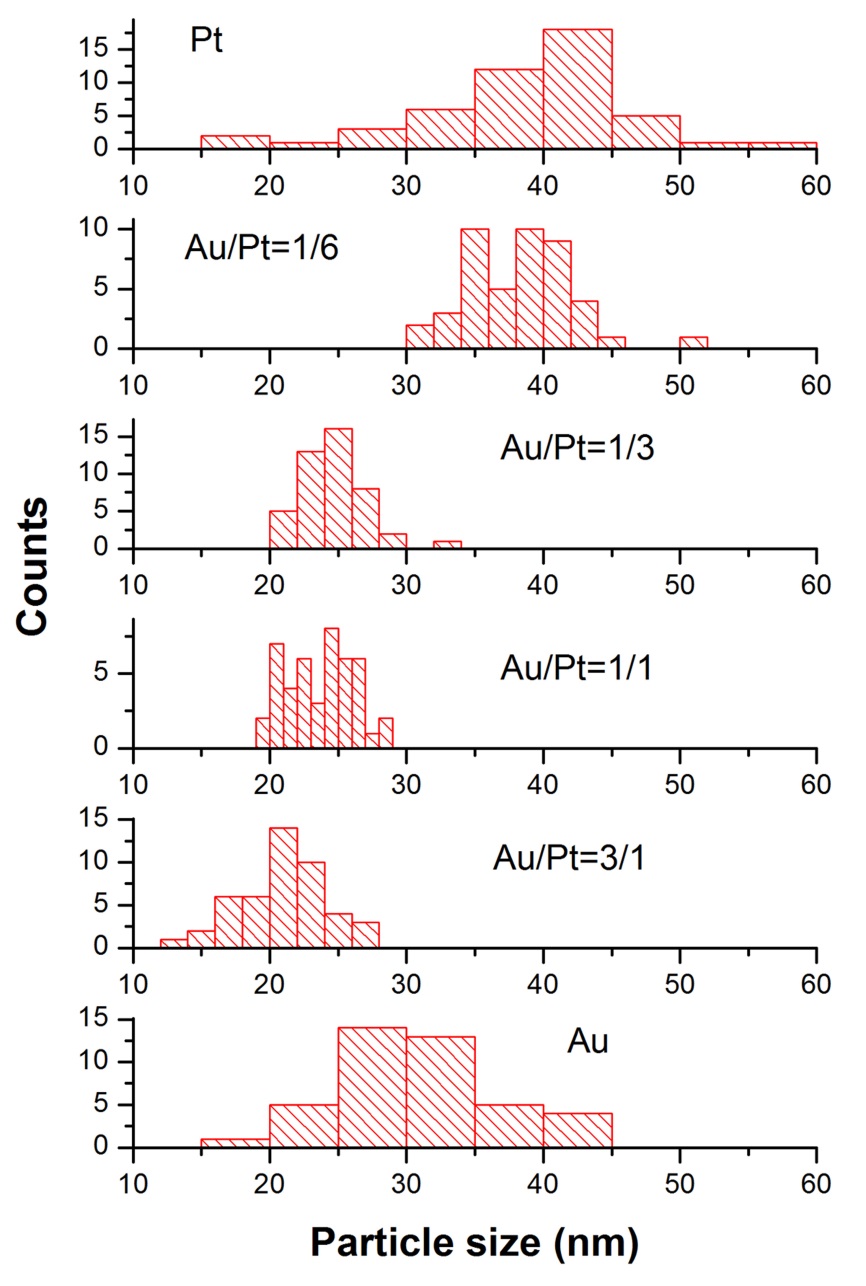


Figure S1. The particle size and size distributions of Au, Pt and AuPt NPs with different Au/Pt ratios.


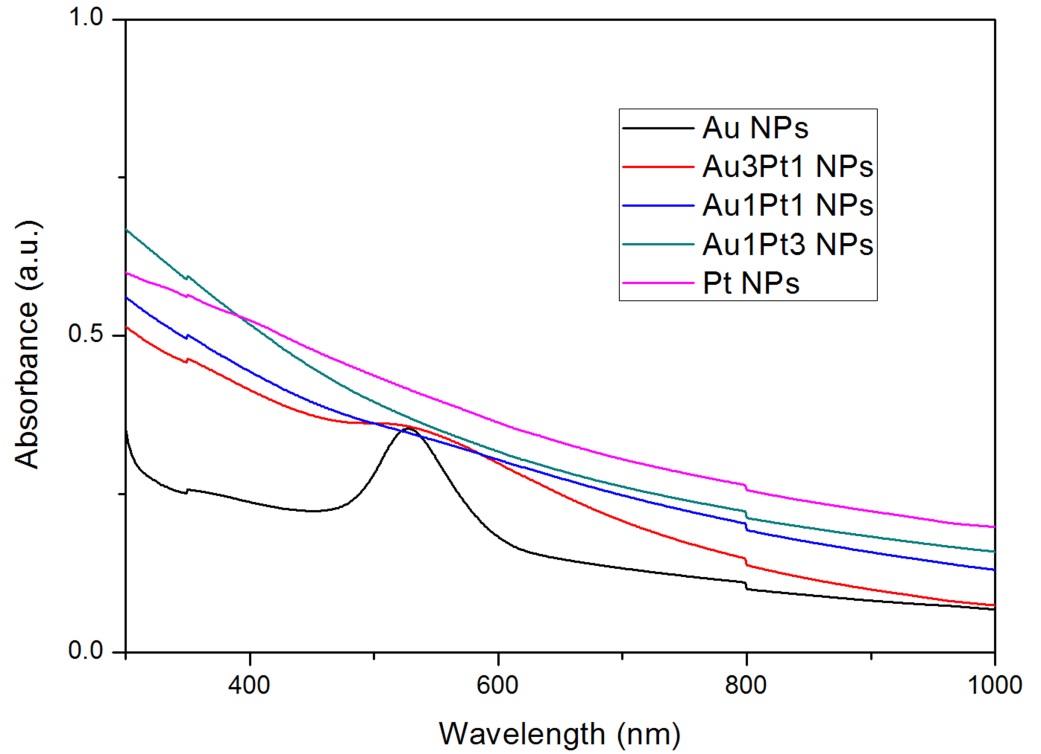


**Figure S2.** UV-Vis spectra of Au NPs, Pt NPs and AuPt NPs having different compositions.


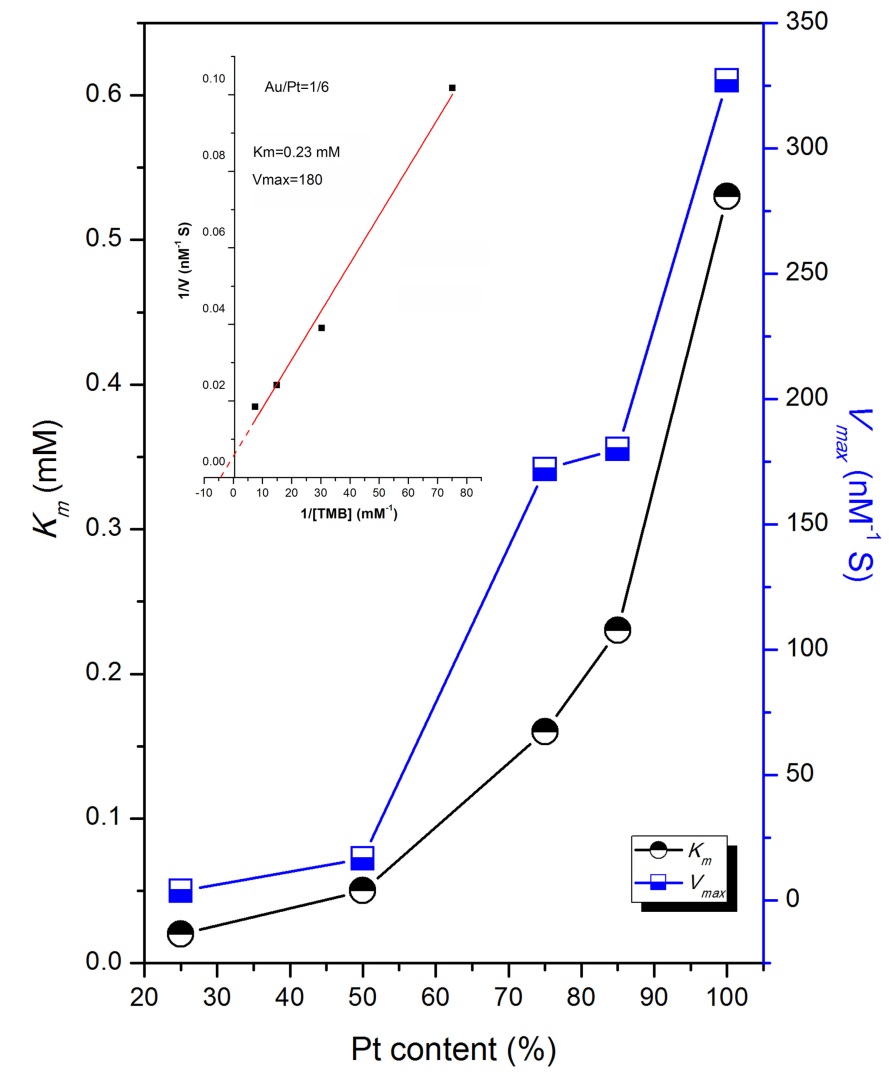


**Figure S3.** Effect of Pt content in AuPt alloy NPs on values of Michaelis constant and maximal reaction velocity for TMB oxidation. The values were found to be dependent on the content of Pt. Inset shows the representative double-reciprocal plots for calculation of enzyme kinetic parameters by the Michaelis-Menten equation.


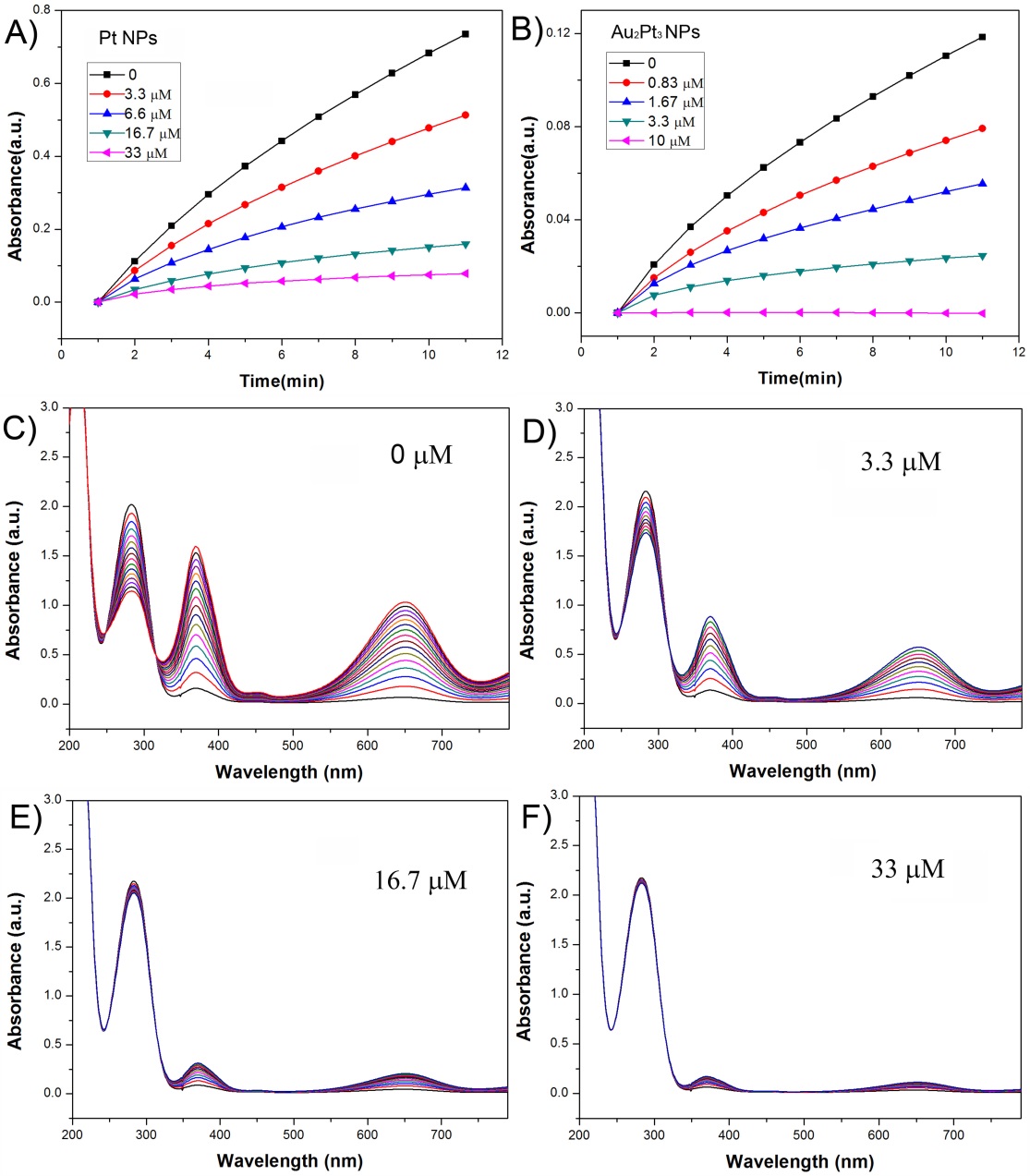


**Figure S4.** Inhibitory effects of HS^-^ ions on the oxidase-like activities of Pt NPs and Au_2_Pt_3_ NPs toward TMB oxidation. HS- ions show concentration dependence on inhibiting the TMB oxidation catalyzed by either Pt NPs (A) or Au_2_Pt_3_ NPs (B). The absorption spectra evolution over reaction time for TMB oxidation catalyzed by Pt NPs in the absence of HS^-^ and different concentration of HS^-^ (C-F).


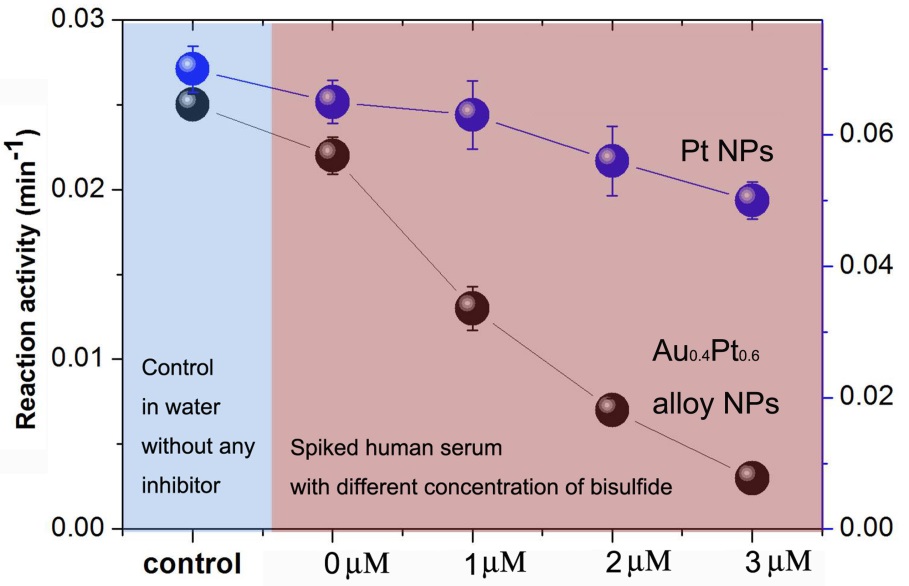


Figure S5. Detection capability for bisulfide in spiked human blood serum by Pt NPs and Au_0.4_Pt_0.6_ alloy NPs.
